# Gastric Volvulus: A Rare Entity Case Report and Literature Review

**DOI:** 10.7759/cureus.2312

**Published:** 2018-03-12

**Authors:** Aisha Akhtar, Fasih Sami Siddiqui, Abdul Ahad E Sheikh, Abu Baker Sheikh, Abhilash Perisetti

**Affiliations:** 1 Department of Surgery, Texas Tech University Health Sciences Center; 2 Student, Florida Hospital-Orlando; 3 Student, Shifa College Of Medicine, Islamabad; 4 Student, Texas Tech University Health Sciences Center; 5 Department of Hospital Medicine, Texas Tech University Health Sciences Center

**Keywords:** gastric volvulus, mesenteroaxial volvulus, small bowel obstruction

## Abstract

Gastric volvulus is a rare entity defined as an abnormal rotation of the stomach around itself. It is a diagnosis of exclusion; the clinical index of suspicion is always low and is mostly diagnosed on imaging or on the surgery table. When it occurs, it is an emergency due to the risk of strangulation and consequent gangrene of the stomach. Mesentero-axial (MA) gastric volvuli constitute one-third of all cases. Here, we are present an interesting case of acute MA gastric volvulus diagnosed with imaging and treated subsequently.

## Introduction and background

Gastric volvulus is a rare entity defined as an abnormal rotation of the stomach around itself. Berti was the first to describe it in 1866 as an autopsy finding, and Berg was the first to successfully treat it surgically in 1897 [1]. Gastric volvulus is characterized by an abnormal rotation of the stomach around its short or long axis leading to variable degrees of gastric inlet and outlet obstruction. A rotation of more than 180 degrees can cause strangulation, necrosis, and finally perforation; hence, it is a surgical emergency. Acute mesentero-axial (MA) volvulus is more common in children and young adults, with few cases reported in the elderly. Due to advances in diagnosis and management, the mortality from acute gastric volvulus is now 15%-20%, and that for chronic gastric volvulus is 0%-13% [2]. Acute idiopathic gastric volvulus in the elderly is a rare entity. We present this case because it is an uncommon life-threatening disease. Given its rarity and unusual presentation, the clinical index of suspicion is always low. Gastric volvulus is mostly diagnosed on imaging or on the surgery table.

## Review

Case

**Figure 1:**
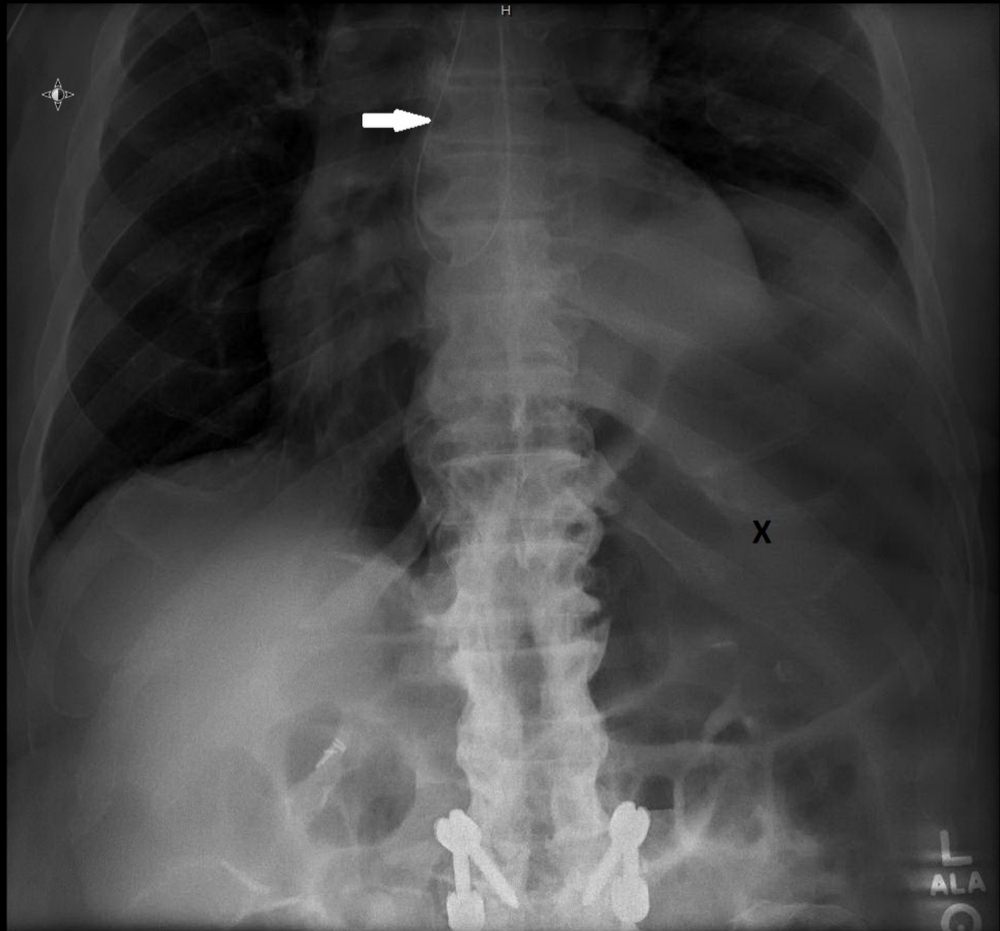
X-ray of the patient showing the nasogastric tube coiled up in the esophagus (white arrow) with a huge distended stomach (letter X)

A 68-year-old male presented for an evaluation of small bowel obstruction. He had a prior medical history of hypertension and chronic pancreatitis diagnosed four years ago and a surgical history of left total knee arthroplasty, followed by a revision three weeks ago (was residing at a nursing home for rehabilitation). The patient reported a one-day history of epigastric pain, generalized abdominal discomfort, distension, along with an eight-day history of constipation. He was passing flatus and also noticed progressive worsening abdominal distension for the past two days with uncontrollable belching and retching, but denied any vomiting or fever. He was given laxative enemas at the nursing home for his constipation and had a subsequent bowel movement, after which he developed abdominal pain. He was taken to a nearby hospital where an X-ray of the abdomen was performed which showed a huge distended stomach with the nasogastric tube (NG) coiled up in the esophagus (Figure 1). Computed tomography (CT) scan of the abdomen showed huge gastric distension with no free fluid or air.

On presentation at our hospital, the patient was still complaining of epigastric pain, retching, and belching. On physical examination, the patient was in slight distress. Oxygen saturation on pulse oximetry was 97%, blood pressure (BP) was 132/75 mmHg while supine, heart rate was 97/min, and the temperature was 97.4 degrees Fahrenheit. He was alert and oriented times three. There was no conjunctival pallor. The pupils were about 4 mm bilaterally and reactive to light. On auscultation, he had dual heart sounds with no murmurs noted. Breath sounds were bilaterally equal with no crackles or rhonchi. The abdomen was soft, non-tender, mildly distended with no masses noted.

**Figure 2:**
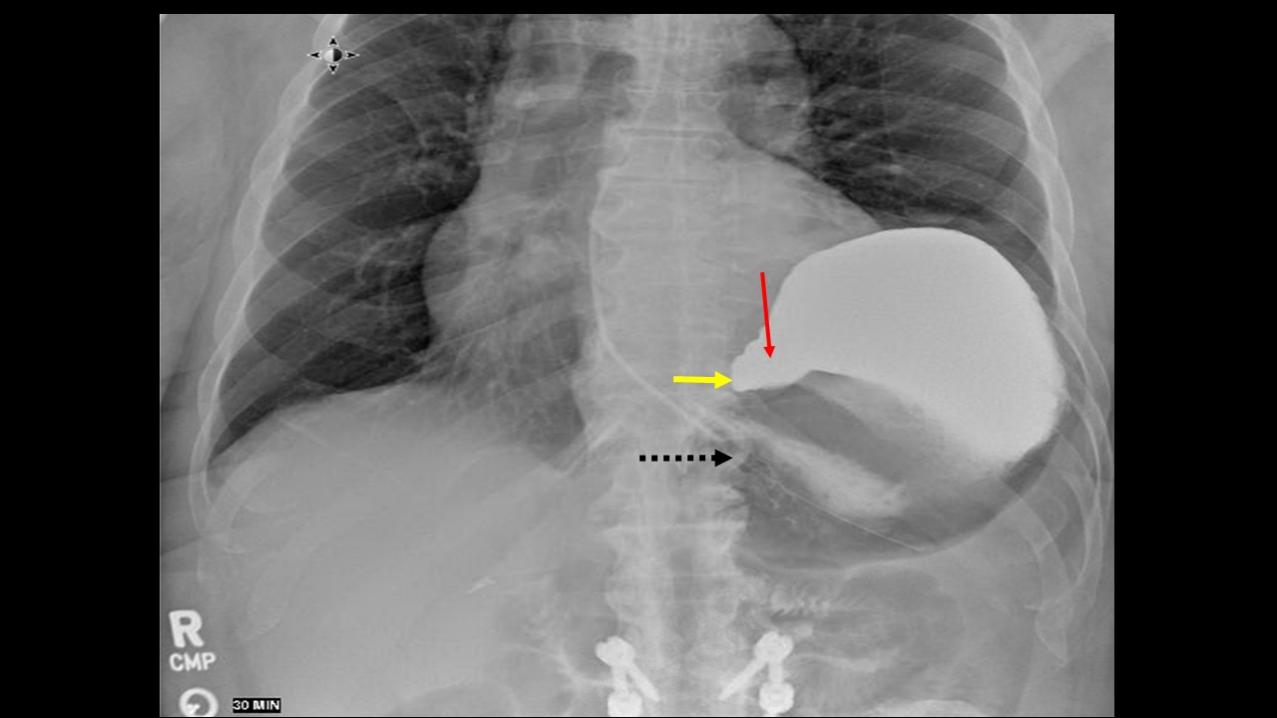
Small bowel of the patient showing the antrum (red arrow) and pylorus (yellow arrow) displaced above the gastroesophageal junction (black dotted arrow)

**Figure 3:**
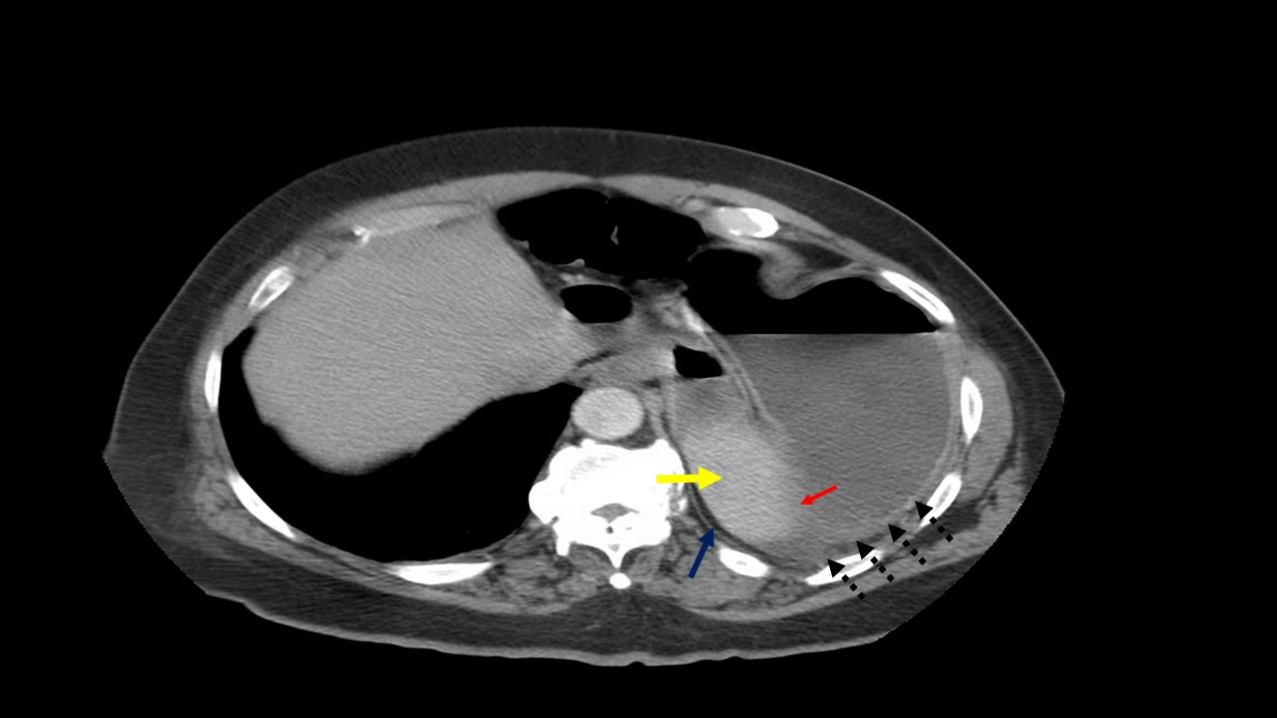
Computed tomography scan of the stomach The stomach appears upside-down with the antrum (red arrow) and pylorus (yellow arrow) superior to the fundus (blue arrow) and proximal body (black dotted arrows).

Laboratory testing revealed a slightly elevated white blood cell (WBC) count of 12.8 K/uL, hemoglobin of 10.5g/dL, and a platelet count of 262,000/uL. Electrolytes were within normal limits. Chest X-ray showed NG in the esophagus which was advanced into the stomach under fluoroscopy. X-ray of the abdomen showed ileus. He was afebrile, non-septic, and treated conservatively for one day, but his symptoms didn’t resolve. A small bowel follow-through study was performed which showed MA gastric volvulus with the antrum displaced above the gastroesophageal junction as best demonstrated in Figure 2. The small bowel showed normal caliber and the fold pattern was unremarkable. Only a small amount of contrast would pass beyond the stomach due to the volvulus. A subsequent CT scan confirmed the displacement of the antrum above the gastroesophageal junction; the stomach appeared upside-down with the antrum and pylorus superior to the fundus and proximal body (Figure 3). He was taken to the operating room where MA gastric volvulus was visually confirmed with the stomach rotated and its greater curvature adherent to the under-surface of the diaphragm. After releasing the stomach from its adherence, gastropexy was done on the right side and a g-tube was placed on the left side. Postoperative recovery was uneventful and the patient returned to his rehabilitation facility on the fourth post-operative day.

Discussion

The term “volvulus” is derived from Latin volvere, which means to turn or roll. Gastric volvulus is a rare entity that was first described by Berti during an autopsy in 1866. It was successfully treated surgically for the first time in 1897 by Berg [1].

Gastric volvulus is characterized by the rotation of the stomach along its short or long axis leading to a variable degree of gastric inlet and outlet obstruction. Rotation more than 180 degrees can lead to strangulation, necrosis, and finally perforation. Hence, it is a surgical emergency. Acute MA volvulus is more common in children; however, a few reports on adults do exist.

Gastric volvulus has been classified based on both etiology (primary or secondary) and the axis of rotation, but the most accepted classification of volvuli is according to the latter as proposed by Singleton [3].

Type I is organo-axial (OA) volvulus (rotation around an axis connecting the pylorus and cardio-esophageal junction; it occurs in 59% of the cases). Type II is (MA) volvulus (rotation around the axis causing bisection of greater and lesser curvature of the stomach; it occurs in 29% of the cases). Type III is a combination of OA and MA (occurs in 2% of cases) and type IV is unclassified (occurs in 10% of cases) [4].

It can also be classified based on etiology as primary/idiopathic volvulus (associated with tumors, adhesions,the or problems in the normal ligamentous attachments of stomach), or secondary volvulus (associated with disorders of gastric motility and anatomy, or with issues of neighboring structures like the diaphragm and spleen) [5]. Most cases of gastric volvulus have a secondary cause. Diseases of the stomach, like peptic ulcers, retract the small curvature and predispose the stomach to MA  axis rotation. In adults, secondary gastric volvulus is usually associated with para-esophageal hernia and traumatic diaphragm injury.

Typically, MA gastric volvulus is very rare in the elderly and is mostly reported in young adults and children. The etiology is idiopathic, with an elevation of the left hemi-diaphragm and a peritoneal adhesion band of unknown origin.

The presentation of gastric volvulus can be acute, subacute, or chronic. The classical Borchardt triad of retching, severe epigastric pain, and inability to pass NG tube hold true for gastric volvulus, most of the time, when it presents acutely like in our case. Subacute presentation is associated with vague abdominal pain [5]. An MA volvulus usually presents as a chronic disease.

Diagnosis of gastric volvulus is challenging due to the non-specificity of the symptoms and rarity of the condition; it is usually achieved radiologically in combination with the clinical presentation. Plain radiographs may show two air-fluid levels in the antrum and fundus, or a single air bubble with no additional luminal gas in the supine position, and a ‘beak’ in the cardio-esophageal region. Abdominal CT scan is very accurate in diagnosing volvulus; it shows an upside-down stomach with the pylorus higher than gastro-esophageal junction. Upper gastrointestinal series is also a useful tool [6]. Teague et al. reported (in a study including 36 patients with gastric volvulus) that barium contrast studies were helpful in 84% of the 25 patients who underwent this investigation [7]. As gastric volvulus is rarely suspected according to the clinical symptoms, barium studies are usually conducted as a first-line investigation, and most patients undergo an abdominal CT scan to confirm the diagnosis.

Initial management is nasogastric decompression to decrease the intragastric pressure followed by surgery to check gastric viability, resect gangrenous portion, and perform de-rotation and gastropexy with or without gastrostomy with repair of secondary factors. Emergent laparotomy is still the most common surgical option for patients with gastric volvulus, though laparoscopic interventions have been described. Surgical reduction with or without gastropexy is the most frequently performed procedure [6].

Jacob et al. reviewed 38 cases of gastric volvulus from 1968 to 2001; he reported that occurrences of gastric volvulus are mainly secondary (75.8%); 52.6% of volvuli observed was OA type and 18.4% was of MA type. Surgery was chosen as the treatment option for the majority of patients (33 out of 38), with conservative treatment reserved only for patients who were unfit to undergo surgery [5].

In our patient gastropexy was done on the right side and a g-tube was placed on the left side; postoperative recovery was uneventful.

We searched the literature and conducted a review of all the cases reported from 1999 to 2018 using PubMed. Details such as demographic data, clinical presentation, type of gastric volvulus, diagnostic test, etiology, treatment, and outcome were obtained. Cases which failed to mention this information were excluded. We found 43 case-reports on this topic which are summarized in Tables 1.

**Table 1:** Literature review of all the gastric volvulus cases reported from 1999 to 2018, utilizing PubMed M: male; F: female; MA: mesentaro-axial; OA: organo-axial; C: combine mesentaro-axial and organoaxial; Dx: diagnosis; Rx: treatment.

PUBLICATION	AGE	SEX	Clinical presentation	Type	Dx	Rx	Etiology	Recovery
Palanivelu [2]	23	F	Pregnant, epigastric pain, vomiting, dyspnea, oliguria	OA	CT	Surgical	Hiatal hernia	Uneventful
Jabbour [4]	23	M	Vomiting, epigastric pain, distension, constipation	MA	CT	Surgical	Idiopathic	Uneventful
Nunes [8]	75	F	Abdominal pain and coffee ground emesis	OA	CT	Surgical	Hiatal hernia	Uneventful
Patel [9]	61	F	Worsening localised left sided burning, substernal chest pain, Nausea, vomiting and dyspnea	OA	CT	Surgical	Paraesophageal hernia	Uneventful
Omata [10]	43	F	Abdominal pain with fullness and vomiting	MA	GI series	Endoscopic reduction then surgery	Idiopathic	Uneventful
Arima [11]	50	M	Acute abdominal pain	MA	CT	Surgical	Idiopathic	Uneventful
Al-Naami [12]	34	M	Epigastric pain, dyspepsia, weight loss	OA	Barium swallow	Surgical	Traumatic diaphragmatic hernia (Gunshot)	Uneventful
Lee [13]	79	F	Recurrent epigastric pain and nausea	MA	CT	Surgical	Idiopathic	Uneventful
Atef [14]	56	F	Epigastric pain with early satiety and postprandial vomiting, dyspnea	OA	Barrium Swallow	Surgical	Bochdalek hernia, Diaphragmatic hernia	Uneventful
LIN [15]	36	M	Massive hemetemesis, postprandial abdominal pain and vomiting	MA	CT	Surgical	Paraesophageal hernia	Uneventful
Masjedizadeh [16]	52	M	Recurrent vomiting	OA	CT	Surgical	Sliding hiatal hernia	Uneventful
Karthikeyan [17]	38	F	Upper abdominal pain >> diagnostic EGD performed >> increased severity of abdominal pain and distension	OA	Laparotomy	Surgical	Iatrogenic (following EGD)	Uneventful
Martinez-Perez [18]	77	F	Acute abdominal pain	OA	CT	Surgical	Hiatal hernia	Died
Kosai [19]	79	F	Incarcerated incisional hernia, abdominal pain, nausea, vomiting	OA	CT	Surgical	Hiatal hernia	Uneventful
Black [20]	70	F	Abdominal pain with nausea	OA	CT	Surgical	Paraesophageal hernia	Uneventful
Altintoprak [21]	19	M	Severe abdominal pain and vomiting	MA	CT	Surgical	Idiopathic	Died
Altintoprak [21]	60	F	Nausea and Vomiting, Inability to urinate	MA	CT	Surgical	Diaphragmatic hernia	Uneventful
Kilincalp [22]	25	M	Severe abdominal pain and retching	MA	CT	Endoscopic decompression	Postural deformity	Uneventful
Lianos [23]	28	F	Acute Severe abdominal pain, nausea, and multiple episodes of bilious vomiting	MA	laparotomy	Surgical	Idiopathic	Uneventful
Jeong [24]	50	M	Acute epigastric pain	MA	CT	Surgical	Idiopathic	Uneventful
Ooka [25]	22	M	Acute abdominal pain	MA	CT	Surgical	Idiopathic	Uneventful
Sultan [26]	51	M	Epigastric pain with Nausea and vomiting	MA	Laproscopy	Surgical	Stomach adherent to a chronically inflamed gall bladder	Uneventful
Kang [27]	22	M	Abdominal pain with nausea and vomiting	MA	Upper GI series	Surgical	Idiopathic	Uneventful
Warren [28]	72	F	Acute nausea, acute abdominal pain, retching, distension	OA	Emergency Endoscopy	Surgical	Hiatal hernia	Uneventful
Larssen [29]	75	F	Acute chest pain and Bradycardia	OA	Laparoscopy	Surgical	Hiatal hernia	Pot-Op low Nutritional intake and dumping syndrome
Larssen [29]	90	M	Hematemesis and fever	OA	CT	Surgical	Hiatal hernia	Uneventful
Larssen [29]	49	M	Acute epigastric pain and hematemesis	MA	CT	Surgical	Hiatal hernia	Uneventful
Nayak [30]		M	repeated episodes of non-bilious vomiting and epigastric pain	MA	CT	Surgical	Congenital diaphragmatic hernia and h/o Blunt abdominal trauma	Uneventful
Tabib [31]	75	M	epigastric pain, nausea, and hematemesis	OA	Barium swallow	Surgical	Hiatal hernia	Uneventful
Chen [32]	83	F	Vomiting, chest discomfort	OA	CT scan	Surgical	Supradiaphragmatic hernia	Uneventful
Ghatak [33]	49	F	Weight loss, mild epigastric pain, heartburn, anorexia	MA	Barium + CT	Surgical	Idiopathic	Uneventful
Fansur [34]	80	F	Dull epigastric ache and postprandial bloating	MA	Barium + CT	Conservative (Patient unfit for surgery)	Morgagni Diaphragmatic Hernia	Uneventful
Kim [35]	49	F	Acute epigastric soreness and vomiting	OA	CT	Surgical	Idiopathic	Uneventful
Germanos [36]	62	M	Acute severe upper abdominal pain	OA	Gatrograffin	Surgical	Hiatal hernia	Uneventful
Yakaryilmaz [37]	26	M	Epigastric pain with Nausea and vomiting	OA	CT scan	Surgical	Hiatal hernia	Uneventful
Iso [38]	86	F	Black tarry Stools	OA	CT	Surgical	Hiatal hernia	Uneventful
Singham [39]	81	F	Acute hematemesis and severe epigastric pain	MA	CT	Expired before surgery	Paraoesophageal hernia	Died
Sung [40]	70	F	Left sided chest pain, non-bilious vomiting and shortness of breath	MA	CT scan	Surgical	Diaphragmatic hernia	Uneventful
Woon [41]	73	M	Atypical chest and epigastric pain	MA	CT	Surgical	Idiopathic	Uneventful
Coulier [42]	79	F	Epigastric and left upper abdominal pain with Nausea and vomiting	MA	CT scan	Surgical	Idiopathic	Uneventful
Testini [43]	51	M	Gastric pain, dyspnea, nausea, vomiting	OA	CT	Surgical	Iatrogenic diahragmatic hernia	Uneventful
Shriki [44]	75	F	Chest pain, SOB, hypotension	MA	CT	Surgical	Paraesophageal hernia	Uneventful
Matsuzaki [45]	81	F	Painless postprandial vomiting	C	Barium swallow	Conservative (surgery refused)	Idiopathic	Uneventful
Our Case	68	M	epigastric pain, generalized abdominal discomfort, distension and constipation	MA	Barium swallow	Surgical	Idiopathic	Uneventful

In our literature review, organo-axial type was reported in 46.5% of the cases and the MA type was reported in 51.1% of the cases. Gastric volvulus due to secondary cause was found in 67.4% of the cases. Surgery was the mainstay of treatment in 90.7% of the patients. Death was reported in only three cases and rest of the patients recovered uneventfully.

## Conclusions

Acute gastric volvulus is a surgical emergency with high morbidity and mortality. The most important factor in diagnosing acute volvulus of the stomach is a high index of suspicion. A constellation of clinical symptoms along with radiological studies helps in making the diagnosis. Emergency laparotomy is needed to prevent serious complications like gangrene and perforation.
